# The impact of male burials on the construction of Corded Ware identity: Reconstructing networks of information in the 3rd millennium BC

**DOI:** 10.1371/journal.pone.0185971

**Published:** 2017-10-12

**Authors:** Quentin Bourgeois, Erik Kroon

**Affiliations:** Faculty of Archaeology, Leiden University, Leiden, The Netherlands; University at Buffalo - The State University of New York, UNITED STATES

## Abstract

The emergence of Corded Ware Groups throughout Europe in the 3^rd^ millennium BC is one of the most defining events in European history. From the Wolga to the Rhine communities start to speak Indo-European languages and bury their dead in an extremely similar fashion. Recent *ancient* DNA-analyses identify a massive migration from the Eurasian steppe as the prime cause for this event. However, there is a fundamental difference between expressing a Corded Ware identity—the sharing of world views and ideas—and having a specific DNA-profile. Therefore, we argue that investigating the exchange of cultural information on burial rites between these communities serves as a crucial complement to the exchange of biological information. By adopting a practice perspective to 1161 Corded Ware burials throughout north-western Europe, combined with similarity indexes and network representations, we demonstrate a high degree of information sharing on the burial ritual between different regions. Moreover, we show that male burials are much more international in character than female burials and as such can be considered as the vector along which cultural information and Corded Ware identity was transmitted. This finding highlights an underlying complex societal organization of Corded Ware burial rites in which gender roles had a significant impact on the composition and transmission of cultural information. Our findings corroborate recent studies that suggest the Corded Ware was a male focused society.

## The Corded Ware phenomenon

The emergence of Corded Ware groups throughout Europe in the early 3^rd^ Millennium BC is one of the most defining periods in European history [1]. From the Wolga to the Rhine, these groups used the same material culture and kept similar practices, in particular funerary rituals. Throughout this vast region the dead were given almost identical burial gifts; men were buried semi-flexed on their right side, heads pointing west and facing south; women on their left side with the head towards the east, facing south. Recent ancient DNA (aDNA) studies of the Corded Ware Culture (CWC) explain its emergence as the result of massive migrations and high individual mobility among steppe populations during the third millennium BC [1–3]. However, it is not self-evident that the homogeneity we perceive is the direct result of migrations [4,5]. There is a fundamental difference between expressing Corded Ware identity and having a specific DNA profile. The former entails the adherence to and sharing of information on world views and ideas, while the latter revolves around physical contact. In this paper we add the exchange of cultural information to the discussion on CWC that is now largely dominated by biological perspectives. By adopting a practice perspective combined with similarity indexes and network representations of 1161 Corded Ware burials throughout north-western Europe, we demonstrate the existence of strongly interconnected Corded Ware communities. Furthermore, we show that men’s burials are far more similar across regions as compared to women’s burials. This corroborates the image of a male focused society as suggested by recent aDNA results [6]. Simultaneously we argue that despite the greater female mobility in life, as shown by isotope analyses [1,7], women’s burial practices are distinctly local. This implies that the male burial practice is the prime vector of cultural information exchange between Corded Ware communities. Our method ultimately allows us to investigate the exchange of cultural information and cultural change, thus providing a complimentary perspective to the more recent biological perspectives.

## Communities of practice in the Corded Ware

In this article, we reconstruct Corded Ware group identity through an analysis of shared practices by assuming that these shared practices indicate so-called communities of practice [8,9]. We argue that Corded Ware groups of the 3^rd^ millennium BC form such communities of practice through their shared burial ritual. Each burial represents the coming together of various people who created and participated in the funeral. The burials are the outcome of negotiations among these participants for whom participating in these discussions also contributed to a notion of the proper manner to bury the dead. These notions could be carried elsewhere to be renegotiated and brought into practice. The overall similarity in Corded Ware funerary rituals and material culture is an emergent property of countless such events in which people established a meaningful agreement on the proper way to bury a deceased individual drawing on their past experiences and accumulated notions of how to do so [10].

We study the presence and positioning of grave goods in Corded Ware graves in various regions to arrive at an impression of the extent to which information on the funerary ritual was shared and put into practice. We argue that the positioning and presence of specific artefacts in these graves relates to notions on the proper way to bury, or rather dress, the dead [11–13]. Such notions are part of a cultural image of death: they entail ideas on what items the deceased might need in death and are entangled with world views [14,15].

The central premise of this article is that the presence and positioning of artefacts in a Corded Ware grave can be expressed and compared mathematically to any other Corded Ware grave to determine the similarity between them. By reifying the same cultural image of death, these groups act out a commonly shared notion of what was considered the proper way to bury a deceased individual. Therefore, an exploration of the similarity between burials can be used to sketch the spatial extent of social interactions and connections that gave rise to the perceived uniformity of the Corded Ware groups. Having emphasised similarities, it is important to note that these burial practices are also prone to conscious and unconscious manipulation over time and across space, because of the continuous renegotiation of practices by actors [8]. A greater disparity between any two graves therefore implies a greater divergence between the partakers in the funerary ritual, be it because of a temporal, spatial or social distance.

## Data & method

**Fig 1:**
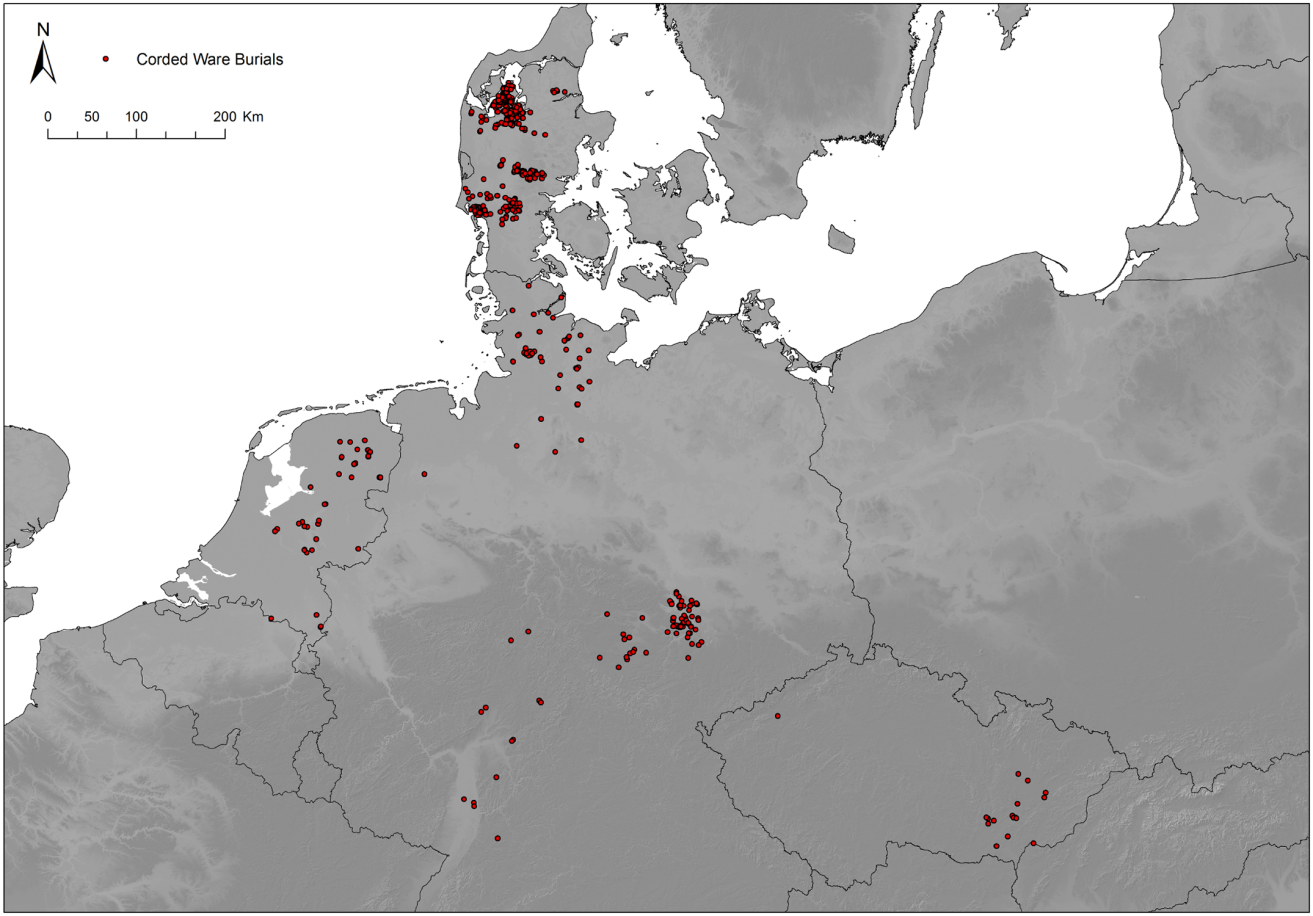
Distribution map of the Corded Ware burials used in the analysis (N = 1161).

In total, we collected data on 1161 Corded Ware burials from well-known burial clusters in north-western Europe as defined by Martin Furholt [16], i.e. Denmark, the Netherlands, Schleswig-Holstein and Niedersachsen, Sachsen-Anhalt, Thuringia, Hessen, Bohemia and Moravia (Fig 1; S1, S2 and S3 Files). For each cluster we selected representative catalogues (see S1 File) and recorded all Corded Ware burials that yielded information on the artefacts *and* their specific position within the burial pit. Note that these selection criteria commonly exclude the majority of the graves in the above-mentioned catalogues.

**Fig 2:**
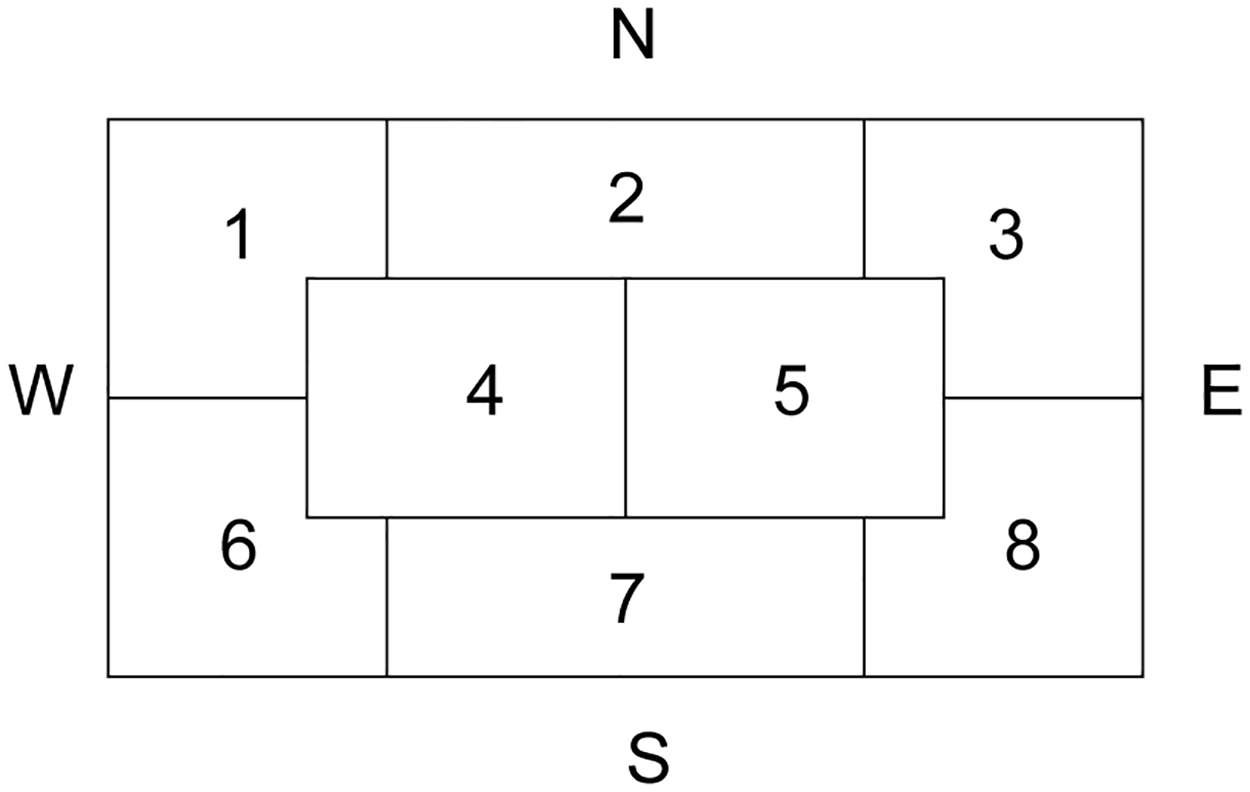
Zoning of a schematic burial pit. This scheme was used to record the position of the different grave goods within each burial. Numbers indicate the code for a specific zone, letters the cardinal directions.

For each burial, we recorded the position of each type of artefact (if any) within the burial pit according to a pre-defined scheme (Fig 2). We scored for 10 artefact categories: the eponymous beakers, battle axes, (flint) blades, amber beads, amber objects (other than beads), flint axes, amphorae, copper ornaments, other pottery (bowls etc.) and a rest category of other objects (shell ornaments, animal teeth, etc.). Note that we define our object categories based on generic classifications rather than typologies that are particular to specific regions to facilitate interregional comparisons. This procedure creates a composite variable that combines the nature of the object with its position in the grave (f.e. a battle axe in the Northwest corner, or a beaker in the northern part of the grave).

Unfortunately, the quality of the data about individual burials varies as not all recorded burials yielded skeletal remains of the deceased due to local preservation conditions. This is a significant problem, because such conditions prohibit statements on the orientation of the deceased and the relation between body and grave goods. On a larger scale, this problem affects the representation of regions where soil conditions are ill-suited for the preservation of skeletal remains (e.g. the Netherlands and the Jutish peninsula; [17]). To overcome these issues, we work with two analytical levels. The first analytical level takes the entire dataset (n = 1161) into account. The second level focusses on two subsets of high quality data and aims to contextualise the patterns observed at the first level. The two subsets of high quality data consist of burials in which the remains of the deceased could be observed. This enables us to make definite observations on the positioning of the body and the placement of the artefacts relative to the body. The two subsets contain the right-flexed (n = 169) and left-flexed burials (n = 112) respectively. In almost all right-flexed burials, the deceased faced South with the head pointing towards the West. The majority of the left-flexed burials forms a mirror image of the right-flexed burials: the deceased faces South and the head points East. It is widely accepted that right-flexed burials are predominantly male burials and that left-flexed burials are predominantly female burials. However, it is important to note that a direct determination of biological sex is often lacking and rare deviations from the norm exist as well [16,18].

The core of our analysis is the calculation of a similarity index for each burial relative to each other burial in the dataset based on the configuration of the grave goods within the burial pit. This calculation is performed through the cosine similarity index which converts each variable into a dimension of a virtual space and then expresses a particular observation as a vector based on the values of these variables. Subsequently, the differences in the angles of these vectors are presented as the degree of similarity between the original entries [19]. We normalised all input to prevent biases based on the number of objects in a grave. The cosine similarity indexes were calculated using *IBM SPSS 23*.

## Reconstructing networks of information

We first aim to establish that Corded Ware burials do indeed exhibit shared practices for placing grave goods in specific places in the burial pit. We then argue that multiple, distinct ways of dressing the dead exist and that these differences primarily relate to the orientation of the body in the grave (i.e. right-flexed versus left-flexed burials). Furthermore, we show that the dissemination of these practices also varies for right-flexed and left-flexed burials.

### Corded Ware funerary practices

**Fig 3:**
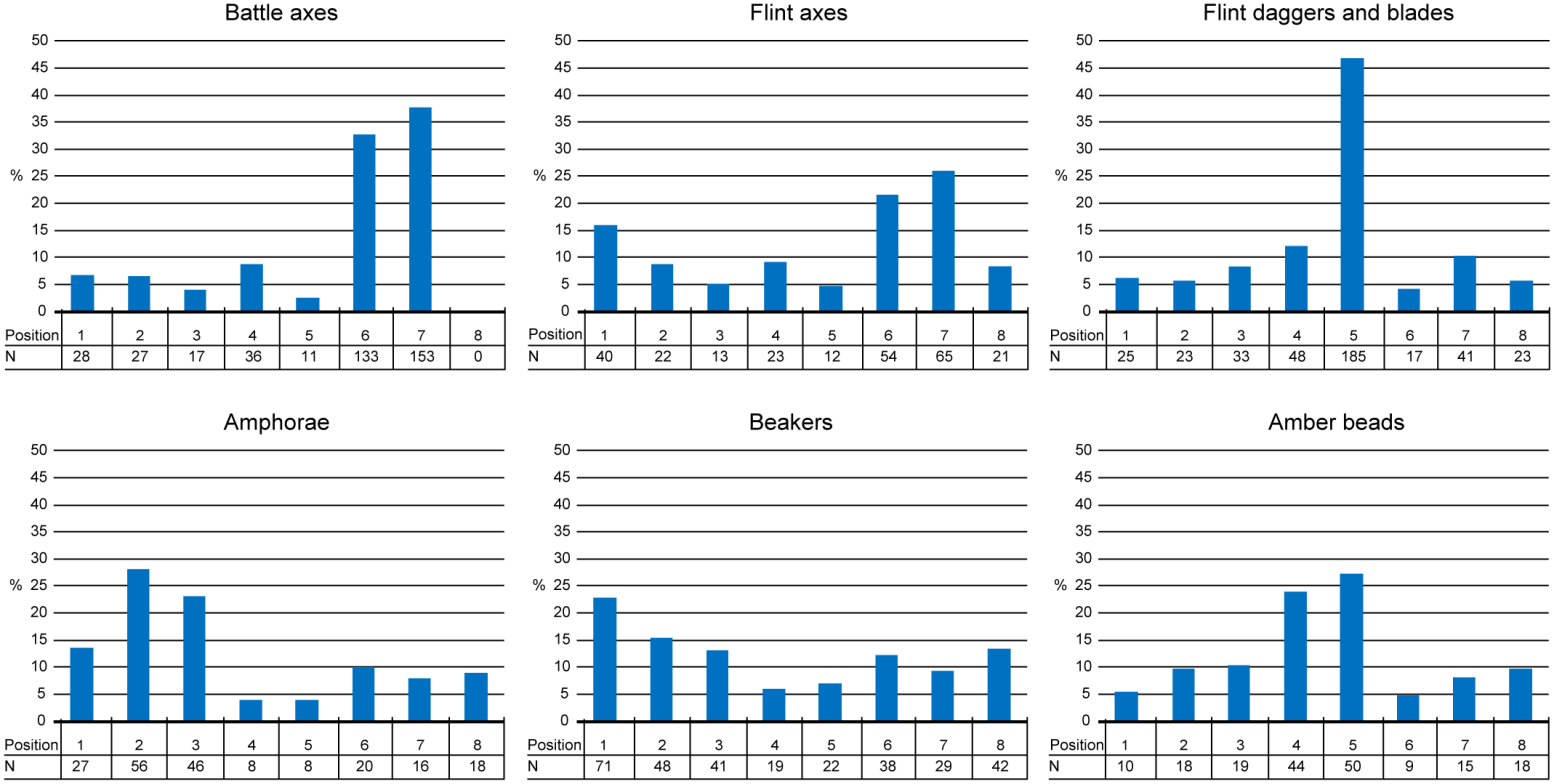
Frequency of artefacts within zones of the burial pit. These histograms present the distributions of specific grave goods (battle axes, flint axes, flint blades, amphorae, beakers and amber beads) across the burial pit as observed in the entire dataset. The tables below the histograms present the absolute amount of objects in a specific position. Despite the temporal and geographical distances between the graves in the dataset, these graphs demonstrate a number of clear preferences in the positioning of grave goods within the burial pit.

As a first step in the analysis we investigated whether or not artefacts were arrayed in relation to the body (Fig 3). In the light of the number of potential combinations of artefact type and position in the grave, it is striking that the majority of the graves from all regions exhibit a base-line of a few very specific arrangements of artefacts relative to the body. This implies that not only wide-spread agreement on proper burial gifts existed, but also strong views on how these artefacts were to be arranged in the grave.

A few practices in the positioning of artefacts stand out within the entire dataset (n = 1161) (Fig 3). One of the strongest practices in the dataset is the placement of battle-axes in the southwest and southern part of the burial pit (322 out of 405 battle axes, 80%). Similarly, flint blades show a strong preference for the centre of the burial pit, specifically the eastern part (185 out of 395, or 47%). Flint axes mirror the battle axes in terms of positioning and both artefacts appear to be interchangeable. Pottery also displays a preferential treatment. Beakers rarely occur in close proximity of the body (41 out of 310, 13%), whereas amphorae tend to be found in the northern and north-eastern edge of the burial pit (102 out 199, 51%).

### Dressing the dead

**Fig 4:**
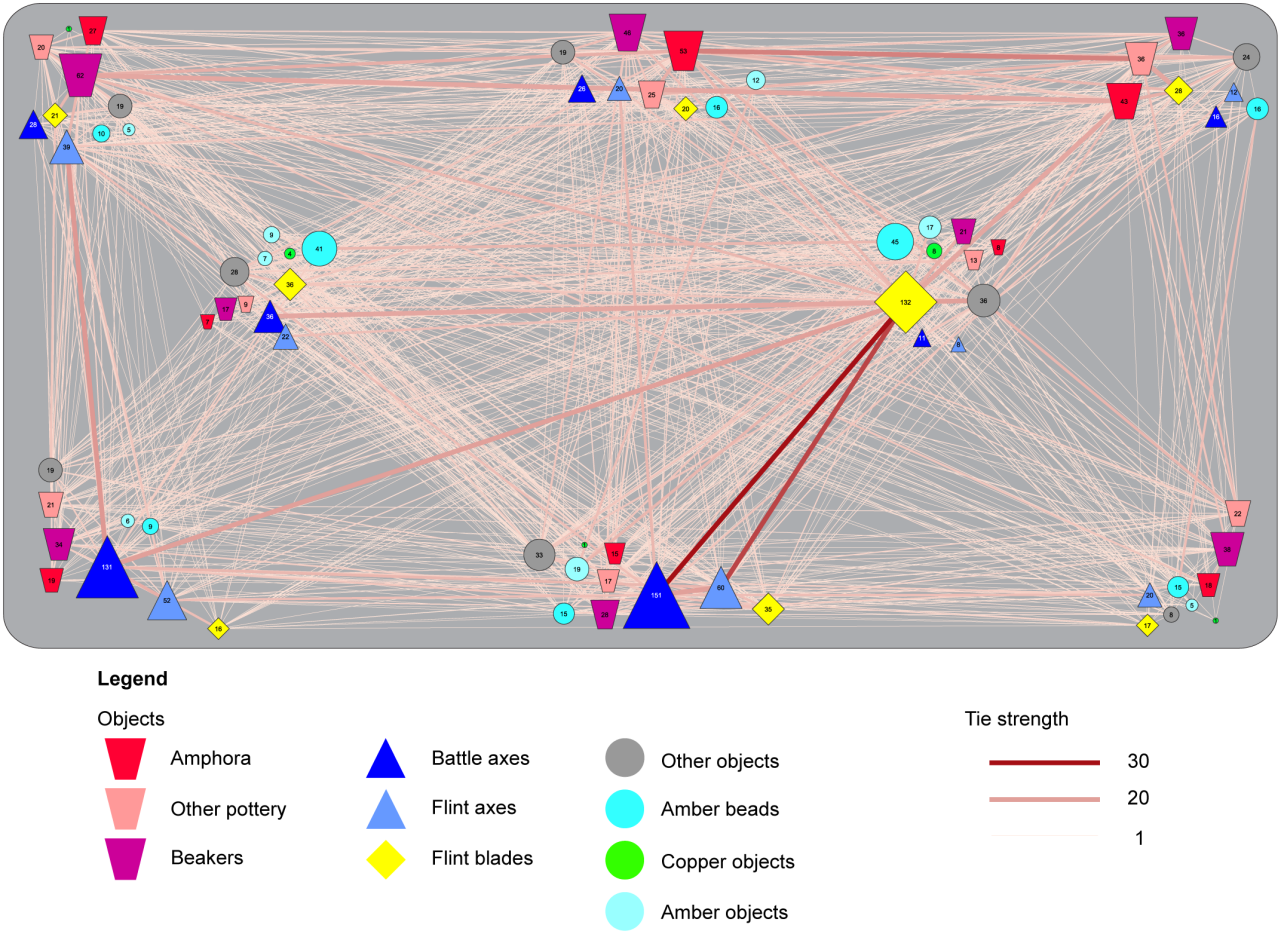
Grave goods position and associations within all burials. This figure is a plot of the relations between grave goods in the entire dataset (N = 1161) against the background of a stylized burial pit. The positions of the symbols correspond to the standardized positions recorded for each individual grave good in the database. The numbers within these symbols show the number of graves that yield this category of grave goods in that particular position. On the one hand, this plot bears witness to the heterogeneity of the dataset as it demonstrates the existence of a multitude of choices in the positioning of artefacts and a large number of relations between these grave goods. On the other hand, it highlights a number of strong correlations and preferences in the positioning of artefacts. Particularly striking is the preferent positioning of flint blades in the centre of the grave and their correlation with flint axes and battle axes at the southern and south-western parts of the burial pit. Furthermore, correlations between different kinds of ceramics are visible at the northern parts of the burial pit.

The positioning of specific artefacts in specific locations already reveals several commonly shared ideas on how to dress the deceased in the grave. This pattern becomes more salient if we plot the co-occurrence of artefacts in specific locations within the entire dataset (n = 1161) (Fig 4). This plot reveals a base-line of strong correlations between specific artefacts that stands out from the heterogeneous data. For example, battle-axes or flint axes placed in front of the body or the face commonly co-occur with flint blades on the pelvis. Pottery is often found at the back of the body and in specific combinations. Within the category pottery, amphorae stand out as these vessels often co-occur with other types of pottery placed at the northern edge of the burial pit. Definite, strong correlations exist between different artefact types in specific locations within the burial pit and some of these correlations mirror patterns that have been described for specific regions [11]. This implies the existence of clear, shared practices in the dressing of the dead.

**Fig 5:**
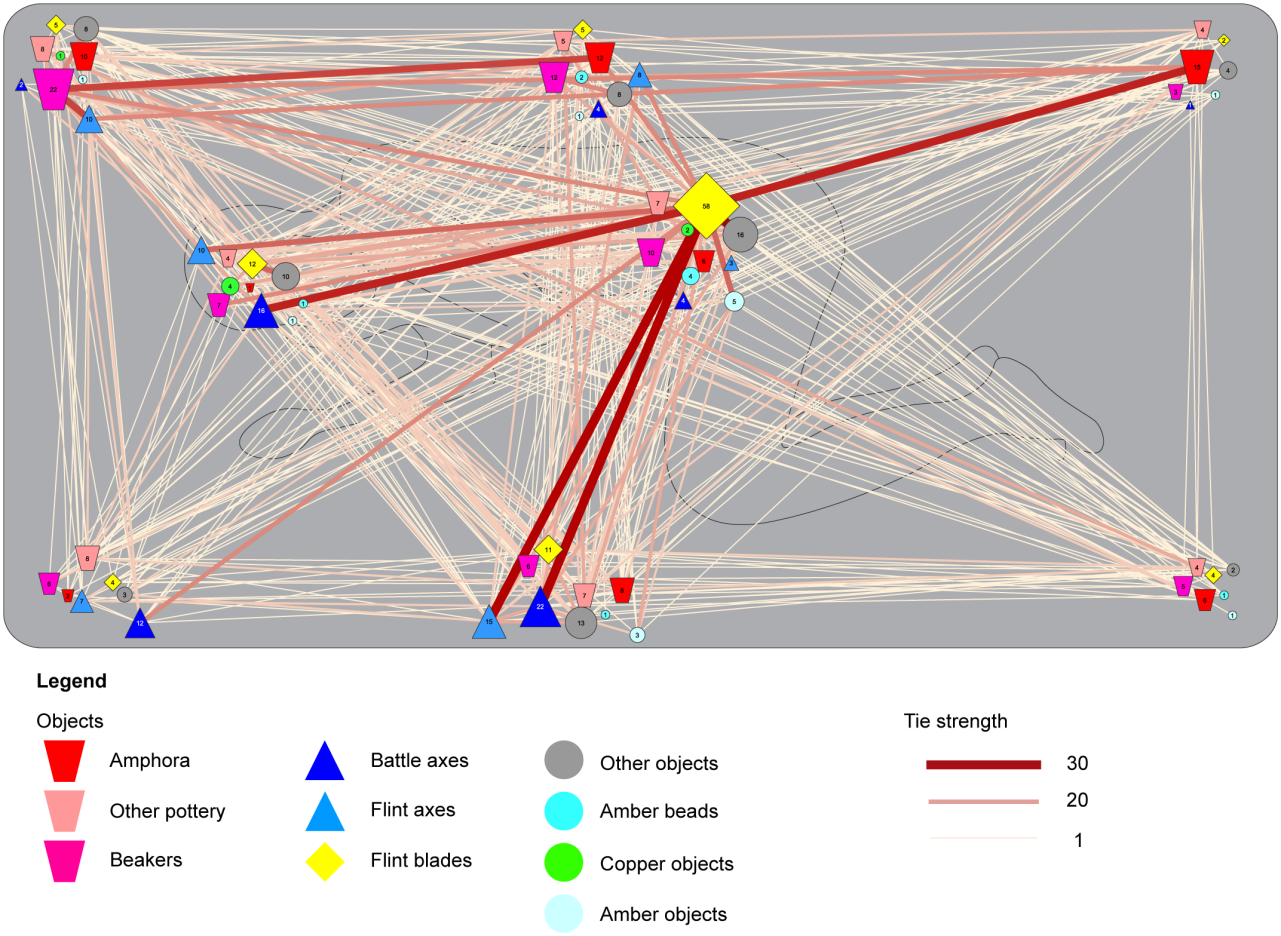
Grave goods position and associations within right-flexed burials. This figure depicts the relations between grave goods in right-flexed burials (n = 169). A stylized burial pit is outlined in grey, a right-flexed body in black lines. The positions of the symbols correspond to the standardized positions recorded for grave goods in the database and the numbers within these symbols are the amount of graves in which that specific object was placed in that specific position. The plot demonstrates that flint blades placed around the pelvic area are a crucial element in these burials. These blades show strong correlations with different types of axes placed in the vicinity of the head or in front of the body (position 4, 6 and 7), as well as with different kinds of pottery that are placed behind the body. Strikingly, the latter two elements share few links, indicating two distinct practices among right-flexed burials.

**Fig 6:**
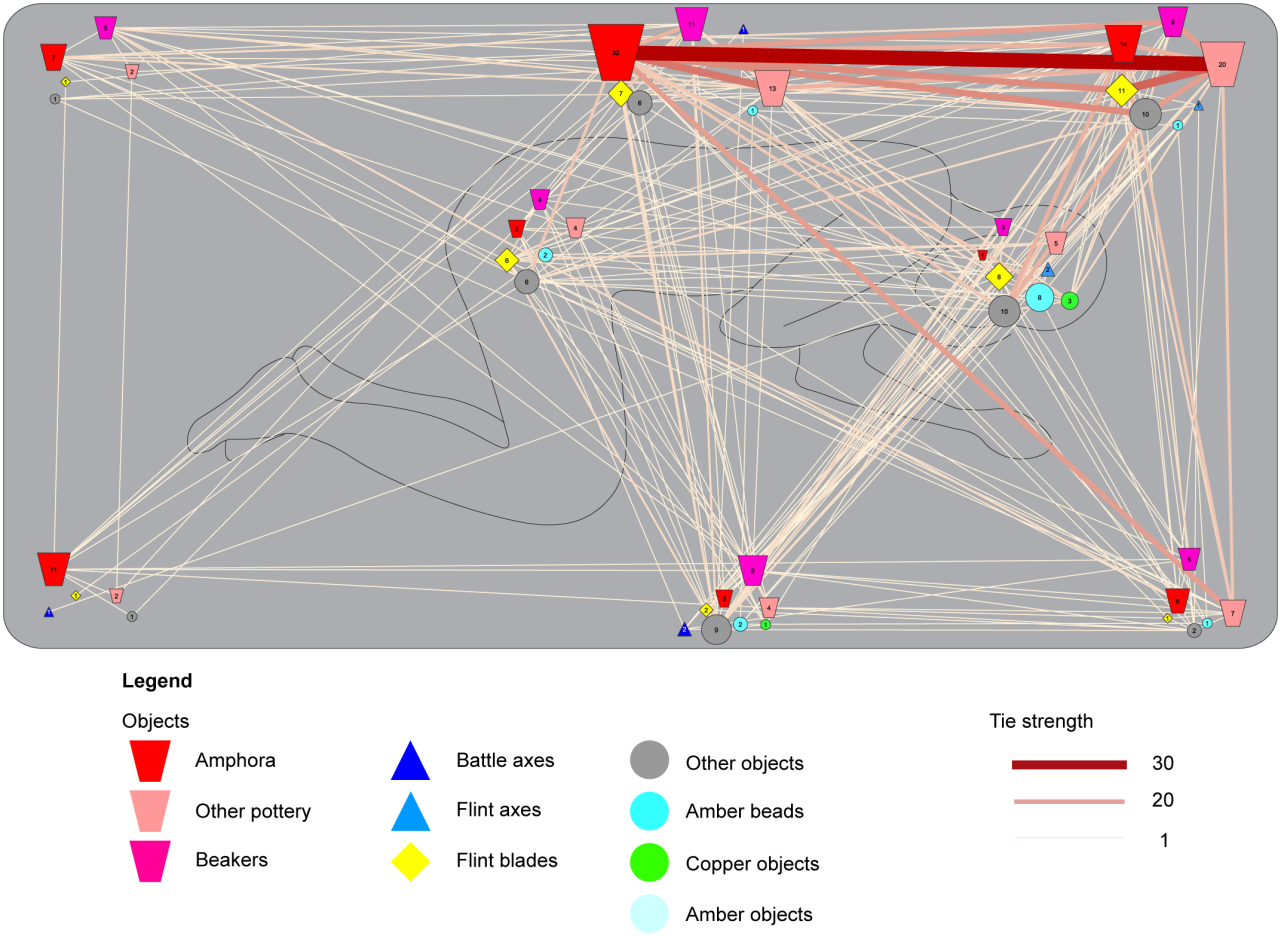
Grave goods position and associations within left-flexed burials. This figure depicts the relations between grave goods in left-flexed burials (n = 112). A stylized burial pit is outlined in grey, a left-flexed body in black lines at the background. The positions of the symbols correspond to the standardized positions recorded for each grave good in the database, whereas the size of the symbol and the numbers within these symbols indicate the amount of graves in which that specific object category was placed in that specific position. In contrast to Fig 4, the majority of the artefacts rarely co-occur with other artefacts. A notable exception is the correlation between different vessel types placed behind the head of the deceased.

For a more detailed view of the role that these practices took in the dressing the dead, we turn to the smaller dataset of burials that yield evidence on the positioning of the deceased (169 right-flexed burials versus 112 left-flexed; Figs 5 and 6 respectively). A key observation is that there are no numeric differences in grave goods between right- and left-flexed burials (median values for number of grave gifts in both groups is 3, and the averages are at 3,67 and 3,07 respectively). An Independent Samples T-test indicates the difference between both groups is not significant (t(279) = 1,081; p = 0,281). Despite the overall similar number of grave goods, left- and right-flexed burials do differ in terms of associated practices. For example, flint blades can be found both in right and left flexed burials. However, these blades are frequently placed around the pelvic area in the former burials (58 out of 101 or 57%), whereas the same item is more often placed behind the head or the back of the deceased in left flexed burials (18 out of 37 or 49%), but rarely on the pelvis (n = 6, or 16%). Battle axes are another case in point. Battle axes are almost exclusively found in right-flexed burials and the majority of these battle axes are placed in front of the body or the face (50 out of 61, or 82%). Conversely, only two battle axes were recovered from left flexed burials. One of these battle axes was placed in the exact same position as battle axes in right-flexed burials, in the southwest corner. In the other burial, the battle axe ended up behind the back of the deceased. Both examples highlight how the orientation of the body seemingly necessitates a differential treatment of similar items.

As with the larger dataset, the co-occurrence of specific artefacts in specific locations within the burial pit is evident from Figs 5 and 6. Several base-line patterns occur in right- and left-flexed burials. A first observation is that left-flexed burials exhibit less correlations between artefacts in specific positions than right-flexed burials. This suggests that left-flexed burials have more disassociated practices (see below).

Right-flexed burials are frequently dressed with a battle axe (or less often a flint axe) in front of the upper body. These axes are commonly associated with a flint dagger placed on the pelvis. Alternatively, a flint dagger in the pelvic area is combined with pottery (beakers and amphorae) behind the body and the head of the deceased. Strikingly, pottery and axes rarely co-occur, hinting at two distinct ways of dressing the dead in the right-flexed burials.

In the left-flexed burials, pottery (amphorae, other types of pottery, and to a lesser degree beakers) is commonly placed behind the back and the head of the deceased. In essence, this a mirror-image of the arrangement of the pottery in right-flexed burials, with the exception that flint blades are commonly placed behind the body instead of on the pelvis.

These observations emphasise that CWC groups did not only share material culture, but also ideas of what to do with these items in burials. If f.e. a battle axe was to be included in the burial, it was supposed to be positioned in front of the body and a different position of a battle axe was only warranted in rare cases. This point exemplifies the differences in the placement of objects between the right- and left-flexed burials. It also explains part of the heterogeneity that we perceive in the larger dataset: certain items merited a different position in relation to the body depending on the orientation (and as such the presumed gender) of the deceased.

### Reconstructing networks of information

Establishing that shared practices exist over such a vast area and time-depth is interesting in its own right. Apparently, multiple clear views existed on the proper construction of a burial, indicating intensive communication between Corded Ware groups. By reconstructing the similarity between burials from different regions we can reconstruct the degree to which these regions shared information on these burial rituals, and as such how the networks of information in the 3^rd^ millennium BC were organized.

In order to achieve this, we took the analysis a step further and compared the build-up of each burial with each other burial in the complete dataset (n = 1161) using a cosine similarity index as described in the methodology section. In this way, the resemblance of each burial to every other burial in terms of the placement of artefacts is expressed on a scale from 0 to 1, with 1 representing identical burials. The resulting similarity matrix allows us to make inferences about the degree of information sharing between regions as shown by the similarity between burials in these regions.

**Fig 7:**
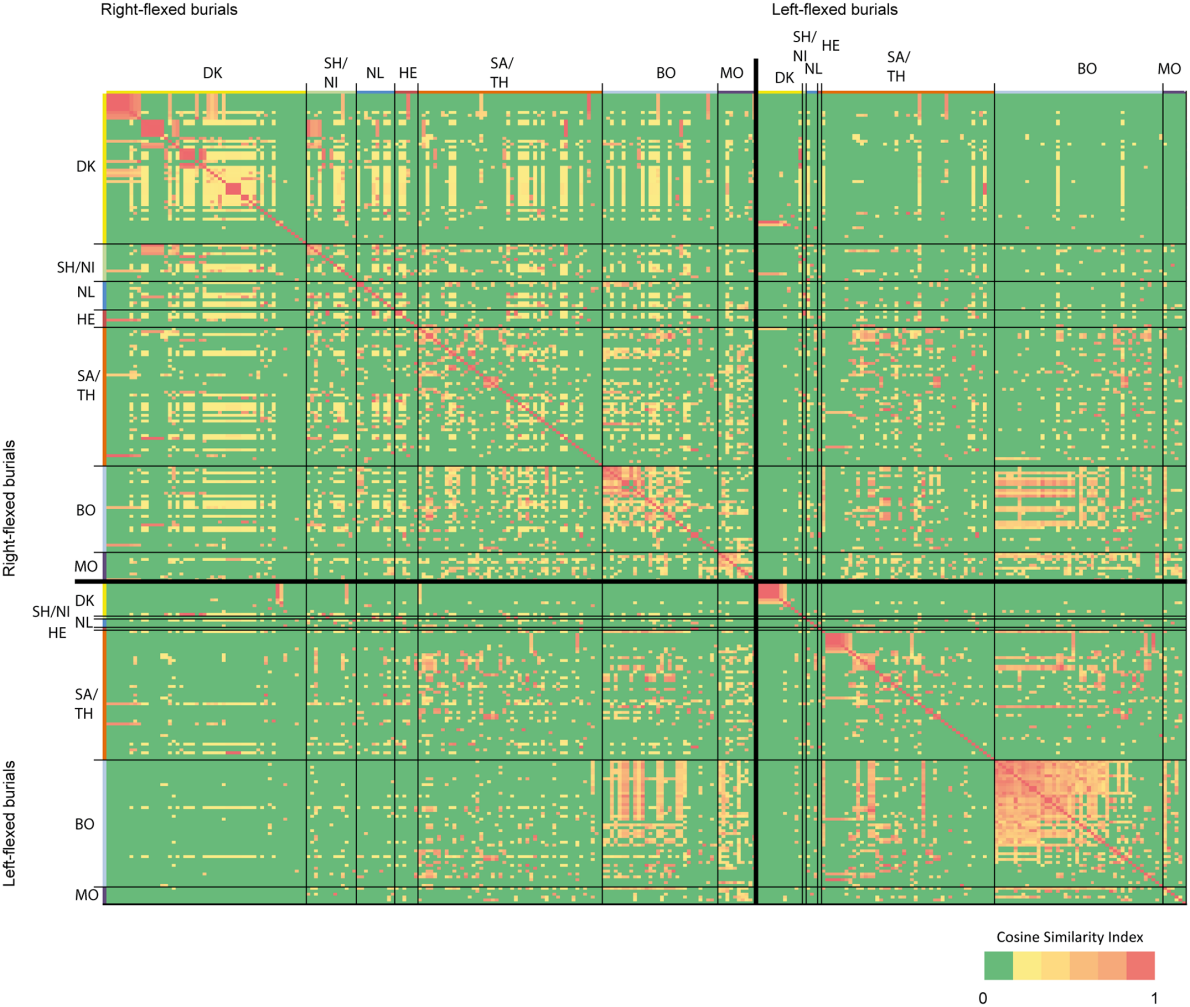
Similarity matrix for all burials that yielded skeletal remains (both right-flexed and left-flexed; n = 281). This matrix expresses the similarity of any two graves in the high-quality dataset on a scale of 0 to 1 (0 for complete disparity, 1 for complete similarity) by means of the cosine similarity index. Axis labels indicate the position of the deceased and the country in which the grave is located. The following abbreviations apply: DK = Denmark, SH/NI = Schleswig-Holstein and Niedersachsen, NL = the Netherlands, HE = Hessen, SA/TH = Sachsen-Anhalt and Thuringia, BO = Bohemia and MO = Moravia. The graves have been sorted on the vertical and horizontal axis based on the values of the cosine similarity index (highest values in top-left corner) and their country of origin for optimal display. The similarity matrix indicates that left-flexed graves are most similar to graves in the same or nearby areas, whereas right-flexed graves display relatively high similarities regardless of geographic regions.

The internal similarity of right flexed burials and left flexed burials within the high quality dataset reveals two different patterns of geographical connectedness (Fig 7). Right-flexed burials exhibit greater similarity to all other right-flexed burials in the dataset regardless of geographical location than do left-flexed burials. Most left-flexed burials bear similarities to burials within the same region. In other words, the right flexed burial ritual contains more elements that are internationally shared than the left flexed burials which are referencing more local norms.

**Fig 8:**
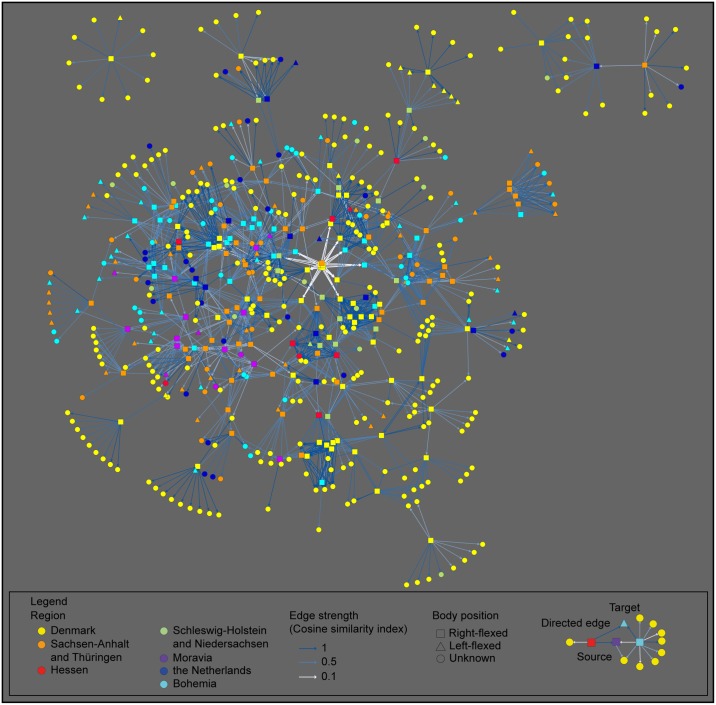
Directed network representation of similarity between right-flexed burials and the overall dataset. The image represents a directed network of the resemblances between each of the 169 right-flexed graves from the high-quality dataset (source) to the ten most similar graves from all 1161 graves (target) regardless of region or bodily orientation in the latter. The similarities are calculated based on the positioning of different grave goods within the burial pit. Most source nodes in this network connect to target nodes from multiple and distant regions, as well as to target nodes in the same or adjacent regions (cf. Fig 10). By implication, the practices for the dressing of the dead right-flexed burials are shared and agreed upon across large distances.

**Fig 9:**
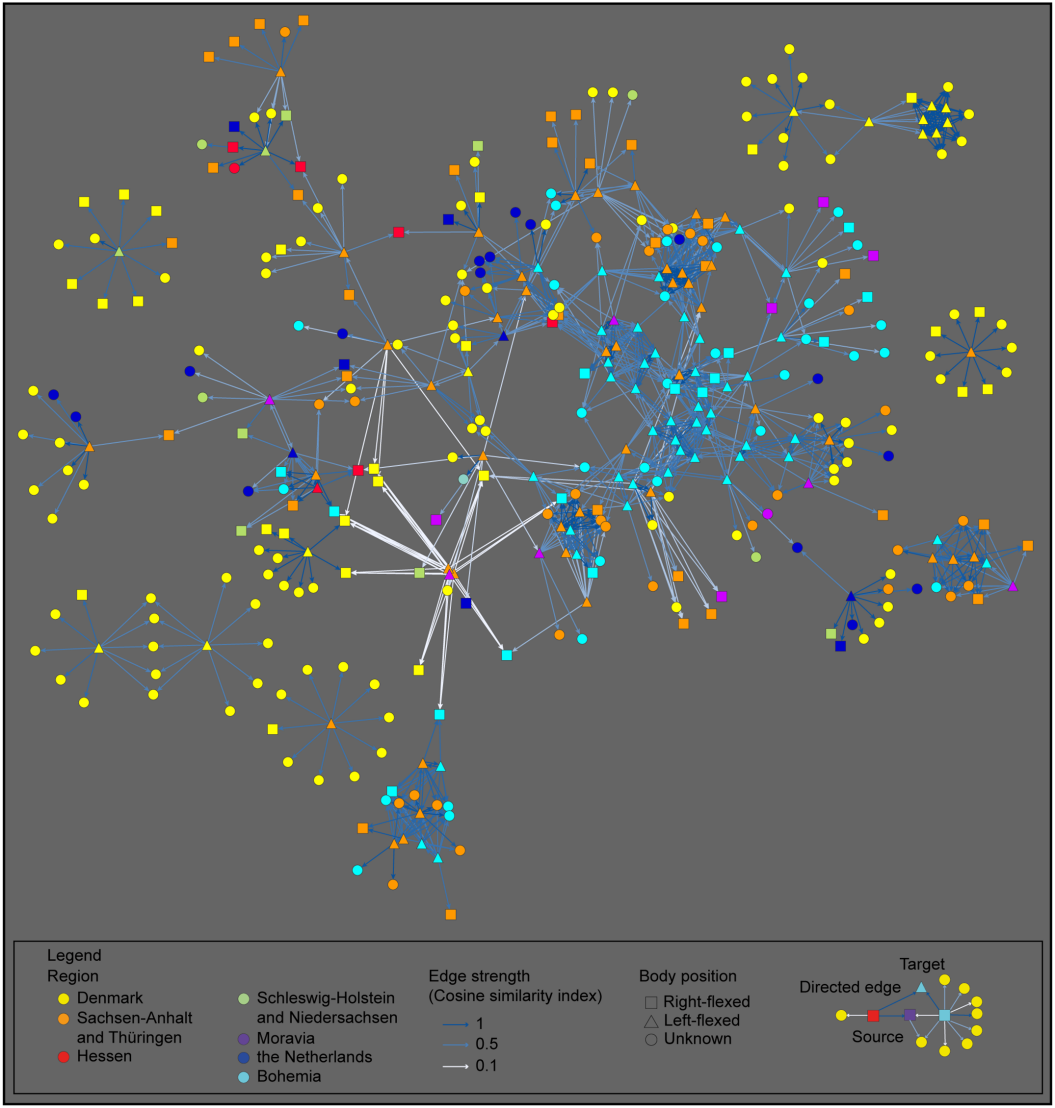
Directed network representation of similarity between left-flexed burials and the overall dataset. The image represents a directed network of the resemblances between each left-flexed grave (n = 112) from the high-quality dataset (source) to the ten most similar graves from the entire dataset of 1161 graves (target) regardless of region or bodily orientation in the latter. The similarities are calculated based on the positioning of different grave goods within the burial pit. Contrary to Fig 9, source and target nodes in this network are frequently situated in the same or adjacent areas. This implies that the practices for dressing deceased individuals in left-flexed burials exhibit a tendency to be more idiosyncratic and locally shared than those for right-flexed burials.

**Fig 10:**
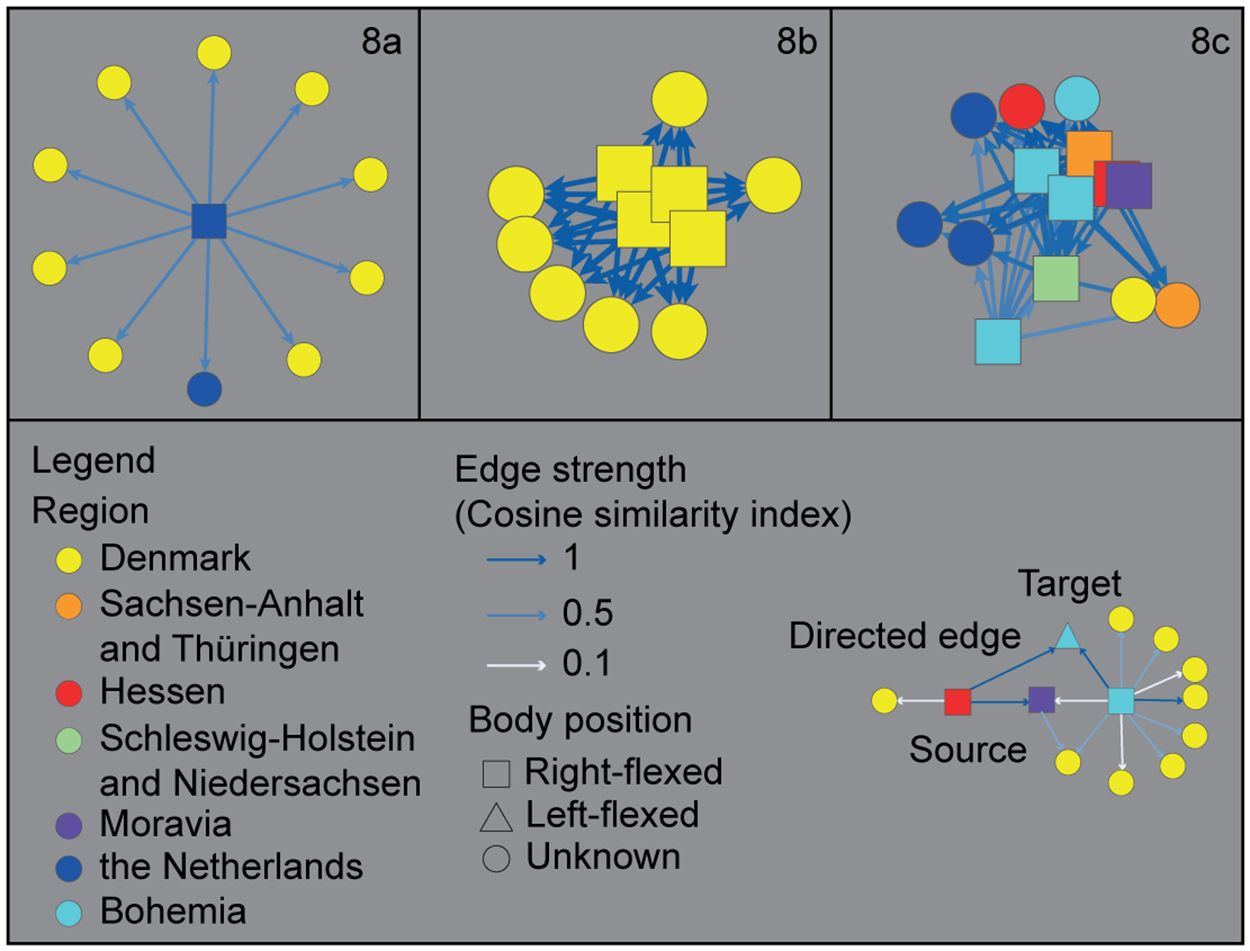
Different scenarios for the connectivity of graves (excerpts from Fig 8). (a) shows a CWC burial from the Netherlands that bears strong resemblances to predominantly Danish graves. In contrast, (b) presents a CWC burial from Denmark whose burial practices appear idiosyncratic to the region as its most similar graves are found exclusively in Denmark. Lastly, (c) can be considered as a supra-regional burial practice with a high degree of similarity between multiple burials and multiple regions.

Given that this matrix of hundreds of burials compared to one another is difficult to visualize, we opted to represent the dataset as a network (Figs 8 and 9 for right- and left-flexed burials respectively). Using the scores of the cosine similarity index as the point of departure, we selected for each of the individual burials in the high-quality dataset (respectively 169 and 112 burials) its 10 most similar burials from the entire dataset (out of a total of 1161 burials). By selecting the 10 most similar burials within the large dataset, we attenuate biases in connectivity caused by regional differences in the data quality related to for example preservation of skeletal remains. The connections between every burial from the high quality dataset to its 10 most similar burials from the entire dataset were then plotted as a network. The strength of each edge in the network reflects the degree of similarity between these burials based on shared practices. In this way, we visualize how specific burials within one region in fact strongly resemble burials from an entirely different region (e.g. a burial from the Netherlands strongly resembles several burials from Denmark, Fig 10a), or inversely, how some burials are entirely idiosyncratic and relate to a specific region and a distinct set of locally informed practices (e.g. a burial from Denmark only has close parallels with other burials from the same region, Fig 10b). The network representation also visualises how certain burials form clusters of shared practices and shared ways of burying (Fig 10c).

Figs 8 and 9 confirm the patterns described above. Left-flexed burials more frequently have equivalent burials within their respective regions (and this is especially true for burials within Sachsen-Anhalt and Bohemia), whereas similarities across regions are more prevalent in right-flexed burials. This implies that the choices people made upon conducting right-flexed burials are adhered to across a wider geographic range than the choices made for left-flexed burials.

## Concluding remarks

The very similar way in which CWC communities created burials and dressed their dead highlights that these communities shared information on this burial ritual over a large area. A person from the Czech Republic attending a funeral in the Netherlands would have recognized and related to many of the actions carried out during the burial ritual. It is one thing to use similar material culture, but our analysis demonstrates that with the use of similar material culture came similar ideas on how to use these items in the burial ritual. This implies that the homogeneity we perceive in Corded Ware society is grounded in intensive communication between its members.

Furthermore, our network representations and similarity indexes demonstrate that women’s burial practices are distinctly local in the CWC. Compared to women, men’s burials are far more similar across regions. By implication Corded Ware communities in different regions were primarily exchanging information on the male burial ritual. Consequently, male burial practices seem to be the prime vector of cultural information exchange between Corded Ware communities.

Our findings corroborate the image of a male focused society that was recently reinstated based on aDNA evidence [1,6,20]. Current theories propose the existence of roaming young male war-bands as one of the core elements in Corded Ware society [1,21]. The international nature of the male burial ritual that we discovered fits this mobility pattern. Intriguingly, the greater female mobility in life as demonstrated by isotopic analyses [1,7], is not reflected in the burial ritual. Concordantly, women’s burials emphasise a more locally rooted information network. The differences in burial ritual and mobility may well shed light on diverging societal perceptions of gender within the CWC.

We argue that studying the exchange of cultural information is a key complement to the more recent biological perspectives on prehistoric migrations and that it provides a unique insight into how prehistoric society was constituted.

## Supporting information

S1 File Supporting data and supporting results. List of all burials used in the analysis as well as the results of the Cosine similarity indexes.
(XLSX)

S2 File Appendix bibliography (part 1). List of all burials used in the analysis and their respective source publications.
(XLSX)

S3 File Appendix bibliography (part 2). Reference list of all publications used in the analysis.
(DOCX)
