# Supplementary material for: The impact of male burials on the construction of Corded Ware identity: Reconstructing networks of information in the 3rd millennium BC
Source: PLoS One. 2017 Oct 12;12(10):e0185971. doi: 10.1371/journal.pone.0185971 (PMC5638321; doi:10.1371/journal.pone.0185971)
Supplement: S3 File — Reference list of all publications used in the analysis. (DOCX) [file pone.0185971.s003.docx]

Arnoldussen S, Scheele EE. The Ancestors Nearby; The domestic and funerary landscape of Angelslo-Emmerhout. In: Velde HM van der, Jaspers NL, Drenth E, Scholte Lubberink HBG, editors. Van graven in de prehistorie en dingen die voorbijgaan. Leiden: Sidestone Press; 2012. pp. 153–185.

Arnoldussen S, Vries KM de. Of farms and fields: the Bronze Age and Iron Age settlement and Celtic field at Hijken-Hijkerveld. Palaeohistoria. 2014;55/56: 85–104.

Beex G. Een Neolithische grafgeuvel met beker en vuursteendolk bij Witrijt (gem. Bergeijk). In: Beex G, editor. Twee Grafheuvels in Noord-Brabant (Bijdragen tot de studie van het Brabantse Heem deel IX). Eindhoven: Brabants Heem; 1957. pp. 7–23.

Bourgeois Q. Monuments on the Horizon; The Formation of the barrow landscape throughout the 3rd an 2nd millennium BC. Leiden: Sidestone Press; 2013.

Buchvaldek M, Koutecký D. Vikletice; Ein Schnukeramisches Gräberfeld. Prague: Universita Karlova (Praehistorica III); 1970.

Bücke S, Barthel H-J, Gall W. Beiträge zur Kultur der mitteldeutschen Schnurkeramiker III. Alt Thuring. 1989;24: 3–116.

Bursch F. Die Becherkultur in den Niederlanden. Oudh Meded uit het Rijksmus van Oudh te Leiden. 1933;14: 39–111.

Bursch F. Een grafheuvel van de bekercultuur te Hoenderloo. Oudh Meded uit het Rijksmus van Oudh te Leiden. 1940;21: 19–22.

Bursch F. Grafheuvelvormen uit het Noorden. Oudh Meded uit het Rijksmus van Oudh te Leiden. 1936;17: 53–72.

Drenth E, Meurkens L. Laat-neolithische graven. In: Hamburg T, Lohof E, Quadflieg B, editors. Bronstijd opgespoord (Archol rapport 142/ ADC rapport 2627). Leiden/Amersfoort: Archol BV/ADC Archeoprojecten; 2011. pp. 197–276.

Drenth E. De inventaris van het graf. In: Williams-Kodde SW, editor. Archeologie op De Woerd; Proefsleuevenonderzoek, archeologische opgraving en archeologische begeleiding aan de Woerdseweg te Groenlo, gemeente Oost Gelre (ADC Rapport 2614). Amersfoort: ADC Archaeoprojecten; 2012. pp. 53–69.

Feustel R, Bach H, Gall W, Teichert M. Beiträge zur Kultur und Anthropologie der mittldeutschen Schnurkeramiker. Alt Thuring. 1966;8: 20–170.

Franke D, Fabian O. Unpublished catalog.

Gebers W. Endneolithikum und Frühbronzezeit im Mittelrheingebiet. Bonn: Rudolf Habelt Verlag (Saarbrücker Beiträge zur Altertumskunde 28); 1978.

Giffen AE van, Addink-Samplonius M, Glasbergen W. Een grafheuvel te Putten. Helinium. 1971;11: 104–123.

Giffen AE van. Die Bauart der Einzelgraber; Beitrag zur kenntnis der älteren individuellen Grabhügelstrukturen in den Niederlanden. Leipzig: Mannubibliothek; 1930.

Giffen AE van. Grafheuvels in Oosterwolde: opgravingen in 1928. De Vrije Fries. 1929;29: 37–60.

Giffen AE van. Oudheidkundige aantekeningen over Drentse vondsten (XVIII). Nieuwe Drentse Volksalm. 1951;69: 97–162.

Giffen AE van. Twee grafheuvels te Nieuw Roden, Gem. Roden. Oudh aantekeningen over Drenthse vondsten. 1935;2: 117–8.

Glazema P. Oudheidkundige onderzoekingen in de provincie Gelderland gedurende de jaren 1946 tot en met 1950. Gelre, vereeninging tot Beoefen van Geldersche Geschiedenis, Oudheidkd en recht Bijdr en Meded. 1951;LI: 1–120.

Grossmann R. Das dialektische Verhältnis von Schnurkeramik und Glockenbecher. Christian-Albrechts-Universität zu Kiel. 2014.

H.R. Reinders, Waterbolk HT, Drenth E. Bewoning in het Neolithicum en de Bronstijd. Nieuwe Drentse Volksalm. 2012;129: 153–184.

Holwerda JH, Evelein, MA. Opgravingen bij het Hanendorp (Emst). Oudh Meded uit het Rijksmus van Oudh te Leiden. 1911;5: 18–22.

Hübner E. Jungneolithischen Gräber auf der Jütischen Halbinsel; Typologische und chronologische Studien zur Einzelgrabkultur. Copenhagen: Det Kongeliche Nordiske Oldskriftselskab; 2005.

Huls RS, Lanting JN, Van der Waals JD. Grabfunde mit frühen Glochenbechern aus Gelderland und Limburg. Ber van Rijksd voor het Oudheidkd Bodemonderz. 1973;23: 77–101.

Jager SW. A Prehistoric route and ancient cart-tracks in the Gemeente of Anloo (Province of Drenthe). Palaeohistoria. 1985;27: 185–245.

Jørgensen E. Hagebrogård - Vroue -Koldkur; Neolithische Gräberfelder aus Nordwest-Jütland. København: Forhistorisk-Arkæolgisk Institut Københavns Universitet; 1977.

Küssner M. Ein reich ausgestattetes Grab der Glockenbecherkultur von Apfelstädt, Lkr. Gotha - Vorbericht. Neue Ausgrabungen und Funde Thüringen. 2006;2: 55–62.

Lanting JN, Mook WG. The Pre- and Protohistory of the Netherlands in terms of Radiocarbon Dates. Groningen: Rijksuniversiteit Groningen; 1977.

Lanting JN, Van der Waals JD. Oudheidkundig onderzoek bij Swalmen. Oudh Meded uit het Rijksmus van Oudh te Leiden. 1974;55: 1–111.

Lanting JN, Waals JD van der. Laat-Neolithische Grafheuvels bij Vaassen en Maarsbergen. Oudh Meded uit het Rijksmus van Oudh te Leiden. 1971;52: 93–127.

Lanting JN. De NO-Nederlandse/NW-Duitse klokbekergroep: culturele achtergrond, typologie van het aardewerk, datering, verspreiding en grafritueel. Palaeohistoria. 2008;49/50: 11–326.

Lipmann E. Schnurkeramische Bestattung von Erfurt. Ausgrabungen und Funde Archäologische Berichte und Informationen. 1982;27: 223–7.

Loewe G. Thüringen; Kataloge zur mitteldeutschen Schnurkeramik. Halle: Landesmuseum für Vorgeschichte, Halle (Veröffentlichungen des Landesamtes für Denkmalpflege und Archäologie Sachsen-Anhalt); 1959.

Matthias W. Kataloge zur mitteldeutschen Schnuerkeramik; Teil IV: Sudhärz-Unstrut-Gebiet. Berlin: Veb Deutscher Verlag der Wissenschaften (Veröffentlichungen des Landesmuseums für Vorgeschichte in Halle 28); 1974.

Matthias W. Kataloge zur Mitteldeutschen Schnurkeramik; T5 Mittleres Saalegebiet. Berlin: Deutscher Verlag der Wissenschaften; 1982.

Matthias W. Kataloge zur mitteldeutschen Schnurkeramik; Teil VI: Restgebiete und Nachträge. Berlin: Veb Deutscher Verlag der Wissenschaften (Veröffentlichungen des Landesmuseums für Vorgeschichte in Halle 40); 1987.

Modderman PJR. Grafheuvelonderzoek in Midden-Nederland. Ber van Rijksd voor het Oudheidkd Bodemonderz. 1954;5: 1–44.

R.N. Halverstad. Het graf. In: Williams-Kodde SW, editor. Archeologie op De Woerd; Proefsleuevenonderzoek, archeologische opgraving en archeologische begeleiding aan de Woerdseweg te Groenlo, gemeente Oost Gelre (ADC Rapport 2614). Amersfoort: ADC Archaeoprojecten; 2012. pp. 51–2.

Šebela L. The Corded Ware Culture in Moravia and in the adjacent part of Silesia (Catalogue). Brno: Archeologický ústav akademie věd České Republiky v Brně; 1999.

Ten Anscher T. Leven met de Vecht: Schokland-P14 en de Noordoostpolder in het Neolithicum en de Bronstijd. Universiteit van Amsterdam. 2012.

Veen M van der, Lanting JN. A group of tumuli on the “Hooghalen” estate near Hijken (Municipality of Beilen, province of Drenthe, the Netherlands). Palaeohistoria. 1989;31: 191–234.

Waterbolk H. Pollenanalytisch onderzoek van twee Noordbrabantse tumuli. In: Beex G, editor. Twee Grafheuvels in Noord-Brabant (Bijdragen tot de studie van het Brabantse Heem deel IX). Eindhoven: Brabants Heem; 1957. pp. 34–9.

Wiermann R. Die Becherkulturen in Hessen. Rahden/Westfalen: Marie Leidorf; 2004.
